# Identification of differentially expressed circulating exosomal lncRNAs in IgA nephropathy patients

**DOI:** 10.1186/s12865-020-00344-1

**Published:** 2020-03-31

**Authors:** Na Guo, Qin Zhou, Xiang Huang, Jianwen Yu, Qianqian Han, Baoting Nong, Yuanyan Xiong, Peifen Liang, Jiajia Li, Min Feng, Jun Lv, Qiongqiong Yang

**Affiliations:** 1 Department of Nephrology, Sun Yat-sen Memorial Hospital, Sun Yat-sen University, Guangzhou, 510000 P.R. China; 2 Department of Nephrology, First Affiliated Hospital, Sun Yat-sen University, Guangzhou, 510000 P.R. China; 3 Key Laboratory of Gene Engineering of the Ministry of Education and State, Key Laboratory of Biocontrol, School of Life Sciences, Sun Yat-sen University, Guangzhou, 510000 P.R. China

**Keywords:** Exosome, Long non-coding RNAs, IgA nephropathy, High-throughput sequencing, Biomarker

## Abstract

**Background:**

Although immunoglobulin A nephropathy (IgAN) is one of the foremost primary glomerular disease, treatment of IgAN is still in infancy. Non-invasive biomarkers are urgently needed for IgAN diagnosis. We investigate the difference in expression profiles of exosomal long non-coding-RNAs (lncRNAs) in plasma from IgAN patients compared with their healthy first-degree relatives, which may reveal novel non-invasive IgAN biomarkers.

**Methods:**

We isolated exosomes from the plasma of both IgAN patients and their healthy first-degree relatives. High-throughput RNA sequencing and real-time quantitative polymerase chain reaction (qRT-PCR) was used to validate lncRNA expression profiles. Pathway enrichment analysis was used to predict their nearest protein-coding genes.

**Results:**

lncRNA-G21551 was significantly down-regulated in IgAN patients. Interestingly, the nearest protein-coding gene of lncRNA-G21551 was found to be encoding the low affinity receptor of the Fc segment of immunoglobulin G (FCGR3B).

**Conclusions:**

Exosomal lncRNA-G21551, with FCGR3B as the nearest protein-coding gene, was down-regulated in IgAN patients, indicating its potential to serve as a non-invasive biomarker for IgAN.

## Background

In Asian populations, Immunoglobulin A nephropathy (IgAN) is known as the most prevalent of primary chronic glomerular disease. IgAN is mostly characterized by the occurrence of IgA1 deposits in the mesangium of glomeruli; usually occurs in young or middle-aged adults. Among the patients, about 20 ~ 40% of cases eventually progress to end-stage renal disease (ESRD) within 10 ~ 20 years [1]. Although the exact mechanism of IgAN is still largely unknown, it is thought to be an immune related disease with a multi-“hit” pathogenetic process with overproduction of aberrantly glycosylated IgA1(galactose-deficient IgA1, Gd-IgA1) [1]. Till now, renal biopsy is still the standard for IgAN diagnosis, with no effective disease-specific therapies currently available. However, renal biopsy is invasive with limitations in assessing disease activity only at the time of biopsy, which could lead to inconclusive findings and decisions [2].

Although a number of IgAN-specific biomarkers have been discussed, the most reported is Gd-IgA1, it has not been validated in large multiracial cohorts of IgAN patients [3]. There are still other studies reported on serum and urinary biomarkers, such as sCD89 [4], the transferrin receptor (TfR) [5], products of complement system (C3b, C3c, C5b-9) [6]. However, there are limitations of these biomarkers, such as the validation in other populations of IgAN patients or lack sensitivity and specificity towards the disease diagnosis and progression. One study identified circulating sCD89-IgA immune complexes was thought about be associated with IgAN disease progression [7]. However, another study measured sCD89-IgA immune complexes in the 326 IgAN patients and found no association with disease progression. With these contradicting results, it was suggested sCD89-IgA is not a good predictor for assessing the progression of IgAN [8]. To date, there are no IgAN-specific biomarkers yet to replace renal biopsy as a diagnostic or to add valuable information in evaluating progression of IgAN revealing the urgent need to identify effective and non-invasive biomarkers to improve early detection and individualized treatment for IgAN.

Long non-coding RNAs (lncRNAs) are known as a heterogeneous class of transcripts with a of more than 200 bases, with no protein-coding potential [9]. Emerging evidence shows that lncRNAs play important roles in various biological processes, including gene expression, protein folding recruitment during chromatin modifications, X-chromosome inactivation/ and immunoregulation [9]. Recent studies have also reported the link between lncRNAs to various kidney diseases [10–13]. Li et al. reported that lncRNA MALAT1 expression was substantially increased in diabetic nephropathy (DN) and could reduce pyroptosis of renal tubular epithelial cells, antagonizing the role of miR-23c on the down-regulation of target gene ELAV like RNA binding protein 1 (ELAVL1), which resulted in a better understanding of the pathogenesis of DN and help the development of new therapeutic strategies [10]. lncRNA X-inactive Specific Transcript (Xist) was identified to be up-regulated in membranous nephropathy (MN). Down-regulation of Xist may improve MN by reducing podocytes apoptosis via miR-217/TLR4 pathway [11]. Liao et al. reported that lncRNA RP11-2B6.2 was increased in kidney tissue from lupus nephritis (LN) patients and positively associated with disease activity and the level of type-I interferon (IFN) [12]. In IgAN patients, Zuo et al. reported 167 differentially expressed lncRNAs (55 up-regulated and 112 down-regulated lncRNAs) while compared with controls, which provided new information on the potential role of lncRNAs in IgAN [13]. Subsequently, this explicit change of lncRNA profile indicated the possibility of lncRNAs to be used as a molecular biomarker for IgAN. However, the inherent instability of lncRNAs (easily degraded by RNase in the blood) results in utterly difficult and inconsistent detection of lncRNAs in the blood is, therefore limits their clinical application [14].

Exosomes are small membrane vesicles with diameters less than 150 nm that are present in nearly all biological fluids (e.g., blood, breast milk, saliva and urine) [15]. Exosomes are known to encapsulate certain proteins, lipids, and RNA, and mediate intercellular communication between various types of cells [15]. Increasing researches are showing that not only can exosomes be diagnostic and prognostic markers for various kinds of malignancies [16], but they also play an important role in immunoregulation and the pathogenesis of immune related diseases such as rheumatoid arthritis (RA) [17], systemic lupus erythematosus (SLE) [18] and IgAN [19]. Exosomes could act as a protective barrier that can protect lncRNAs from extracellular degradation [14, 15], hence could be used as an excellent biomarker to detect significant changes in lncRNA profile in IgAN patients. In recent years, high-throughput RNA sequencing (RNA-seq) has become an attractive choice for the identification of differentially expressed genes (DEG) in various diseases as it has covers most of genome and exact detection of even those low-expressing genes. RNA-seq could achieve the resolution of a “single-base” and capture all transcripts [20]. This study utilized RNA-seq to search for differentially-expressed blood exosomal lncRNAs between IgAN patients and their first-degree healthy relatives. In addition, real-time PCR (qRT-PCR) was also conducted to validate the results of RNA-seq, which may provide clues to identify potential novel lncRNA biomarkers for IgAN.

## Results

### Subject characteristics

In this study, we recruited 17 IgAN patients and their healthy controls (their healthy first-degree relatives). The study was divided into two phases: (i) the screening phase was composed of 6 patients and 6 healthy controls; and (ii) the validation phase, with 11 patients and 11 healthy controls. The demographic and clinical data of subjects were summarized in Table 1. There were no significant differences in age, sex, BMI and blood pressure between patients with IgAN and the controls. However, IgAN patients presented higher 24-UPE (1.14 ± 0.51 vs. 0.00 g/24 h), sCr (94 ± 34.02 vs. 67.00 ± 19.42 μmol/L, *p* = 0.007), UA (422.0 ± 118.0 vs. 341.7 ± 86.16 μmol/L, *p* = 0.030), BUN (6.01 ± 2.90 vs. 4.64 ± 1.31 mmol/L, *p* = 0.086) and lower eGFR (84.66 ± 36.76 vs. 125.89 ± 53.90 ml/min/1.73m^2,^*p* = 0.014) as compared to healthy controls.

**Table 1:** Demographic and clinical characteristics of IgAN patients and healthy controls

Variables	IgAN (*n* = 17)	HC (*n* = 17)	*p* value
Gender (male/female)	8/9	9/8	0.732
Age (Years)	30.76 ± 7.23	38.47 ± 15.79	0.070
Body mass index (Kg/m2)	23.02 ± 3.31	22.53 ± 4.41	0.716
Systolic blood pressure (mmHg)	117.35 ± 18.83	120.29 ± 13.71	0.606
Diastolic blood pressure (mmHg)	72.00 ± 11.83	76.82 ± 7.01	0.158
Mean arterial pressure (mmHg)	87.11 ± 13.71	91.31 ± 8.54	0.292
Urinary protein excretion (g/24 h)	1.14 ± 0.51	ND	ND
Serum uric acid (μmol/L)	422.0 ± 118.0	341.7 ± 86.16	0.030
Serum creatinine (μmol/L)	94.53 ± 34.02	67.00 ± 19.42	0.007
Blood urea nitrogen (mmol /L)	6.01 ± 2.90	4.64 ± 1.31	0.086
eGFR (ml/min/1.73 m2)	84.66 ± 36.76	125.89 ± 53.90	0.014

*Abbreviations*: *eGFR* estimated glomerular filtration rate, *HC* healthy controls, *ND* No Data

### Identification and characterization of plasma exosomes

**Fig. 1:**
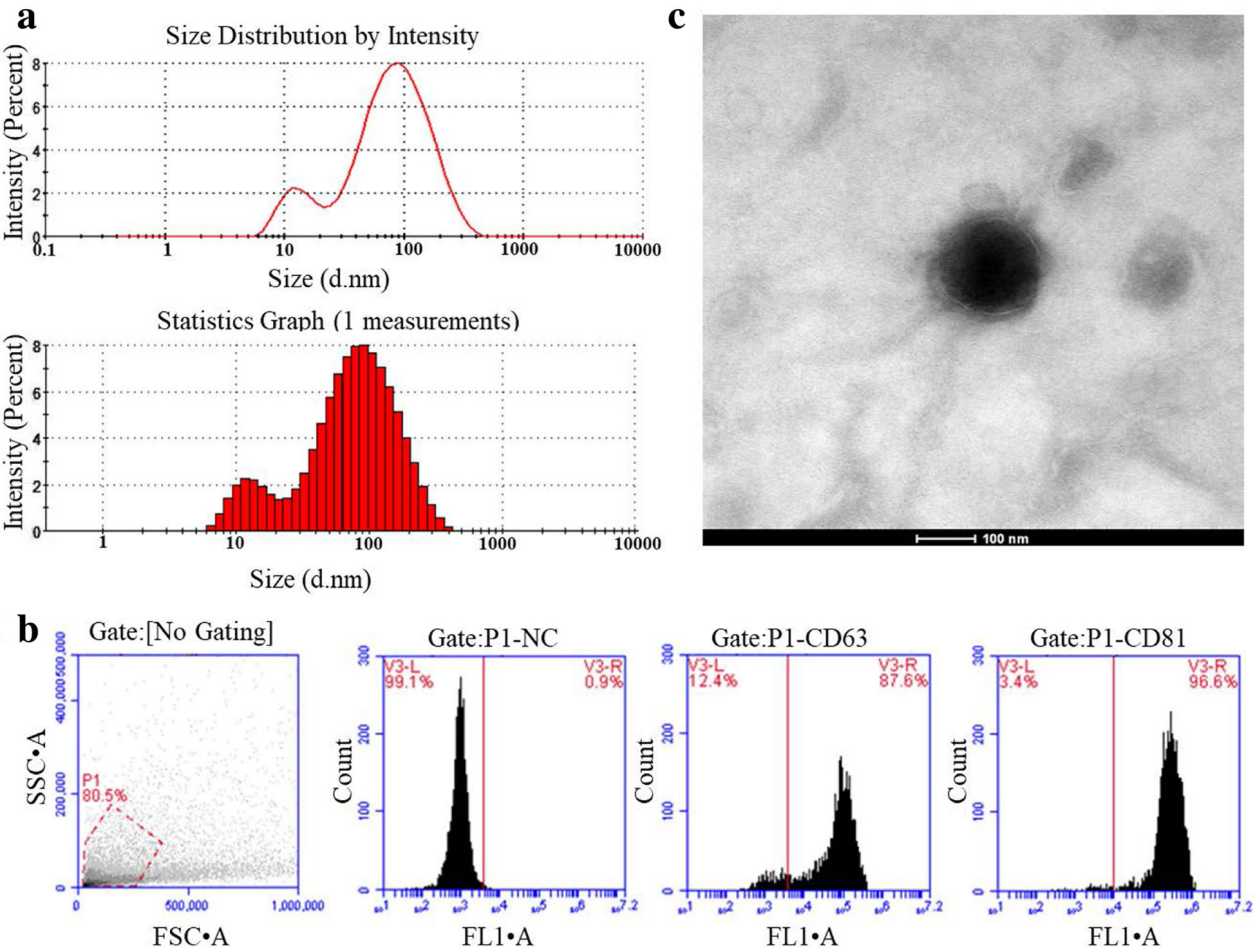
Characterization of exosomes isolated from plasma. **a** The distribution of exosome size by DLS analysis. **b** Flow cytometry analysis of the exosomal surface markers CD63 and CD81. **c** Transmission Electron Microscope (TEM) images of isolated exosomes. Scale bar, 100 nm

Exosome Precipitation Solution (ExoQuick-TC, System Biosciences) was used to isolate exosomes from plasma. After exosome isolation and purification, ZETASIZER Nano series-Nano-ZS (Malvern Instruments Ltd., Malvern, UK) was used to determine the hydrodynamic size of the exosomes. As shown in Fig. 1a, the size of exosome was ~ 30–200 nm. Flow cytometry analysis detected high exosomal surface marker proteins CD63 (87.6%) and CD81 (96.6%), respectively, confirming the purity of exosomes (Fig. 1b). TEM was also performed to determine the physical morphology and size of the exosomes, as seen in Fig. 1c, TEM image showed the lipid bilayer membrane at approximately 100 nm.

### Identification of differentially-expressed lncRNA profiles

**Fig. 2:**
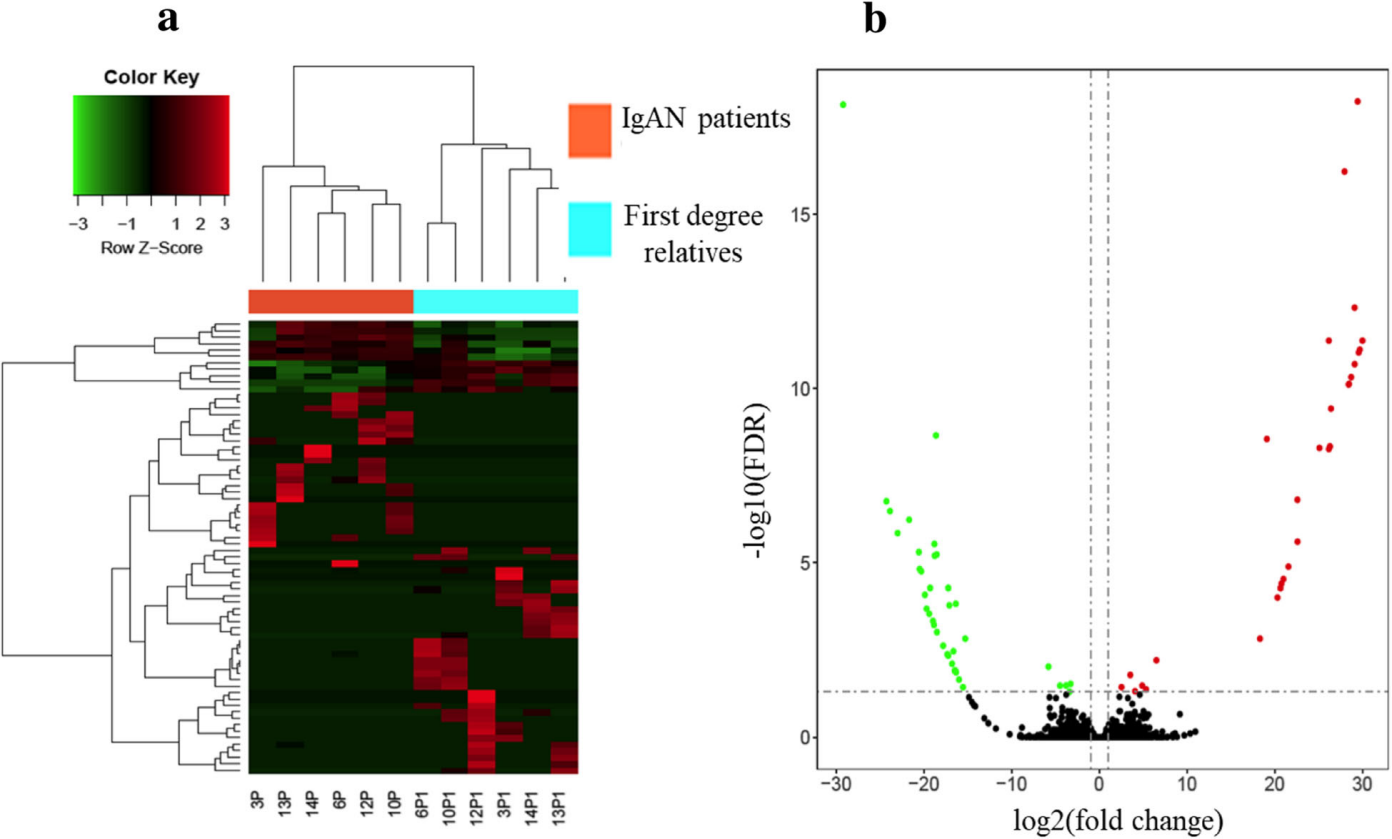
Differential expression profile of lncRNA in IgAN patients of RNA-seq (*n* = 6) and relatives (n = 6) of RNA-seq. (**a**) Heatmap of 70 lncRNAs that are differentially expressed between the two groups. The row z-score depict the lncRNAs expression values (**b**) Volcano plot of the differentially expressed lncRNAs between the two groups, the green, red and black dots represent down-regulated, up-regulated, and non-significance lncRNAs respectively. Cutoff: FDR < 0.05, fold change > 2

RNA-Seq (Illumina, Sna Diego, TX, USA) was performed to characterize the lncRNA expression profiles of exosomes in plasma samples of patients with IgAN and healthy relatives. As shown in Table 2, 70 lncRNAs were differentially expressed with significant fold-change (|log_2_(FC)| > 1) and base mean values. Among the 70 lncRNAs, 31 lncRNAs were upregulated and 39 lncRNAs were down-regulated in the IgAN group compared to control. The heatmap of differential expression and hierarchical clustering of lncRNAs in plasma samples of patients with IgAN and their corresponding relatives was demonstrated in Fig. 2a, while the volcano plot of differential expression of lncRNAs was showed in Fig. 2b.

**Table 2:** Top 10 differently expressed lncRNAs in IgAN patients as compared with healthy controls (sorted by base mean and |log2(FC)|)

ID	Base Mean	|log2(FC)|	*P*adj	Protein-gene
Up-regulated				
G92245	34.51	3.53	0.016	MED13L
ENSG00000234793.1_3	21.57	5.33	0.042	DTYMK
G287980	14.96	4.89	0.033	C4orf45
lnc-FGL2–4	13.28	4.07	0.048	FGL2
lnc-RADIL-1	5.87	29.56	< 0.001	RADIL
lnc-PAXIP1–9	4.98	29.94	< 0.001	PAXIP1
G150385	4.83	19.10	< 0.001	ALOX12
lnc-GALNT2–1	3.24	29.44	< 0.001	GALNT2
lnc-CSTF3–4	3.02	25.09	< 0.001	CSTF3
G316071	2.88	26.41	< 0.001	IL17A
Down-regulated				
lnc-SPATA31E1–10	20.04	3.79	0.033	AL353572.3
G386979	19.67	19.69	< 0.001	SCAI
G36922	16.64	4.48	0.033	PITRM1
ENSG00000248266.1_4	11.67	5.80	0.009	TENM3
lnc-LMTK3–1	6.07	22.99	< 0.001	LMTK3
ENSG00000268605.1_4	5.28	18.96	< 0.001	LIPE
G122951	4.82	18.62	< 0.001	RAB11A
G21551	4.20	29.20	< 0.001	FCGR3B
lnc-REV3L-2	3.61	16.36	< 0.001	REV3L
G111779	1.22	24.27	< 0.001	GTF2A1

|log2(FC)|: |log2(fold-change)|; Padj: adjusted *p*-value

**Fig. 3:**
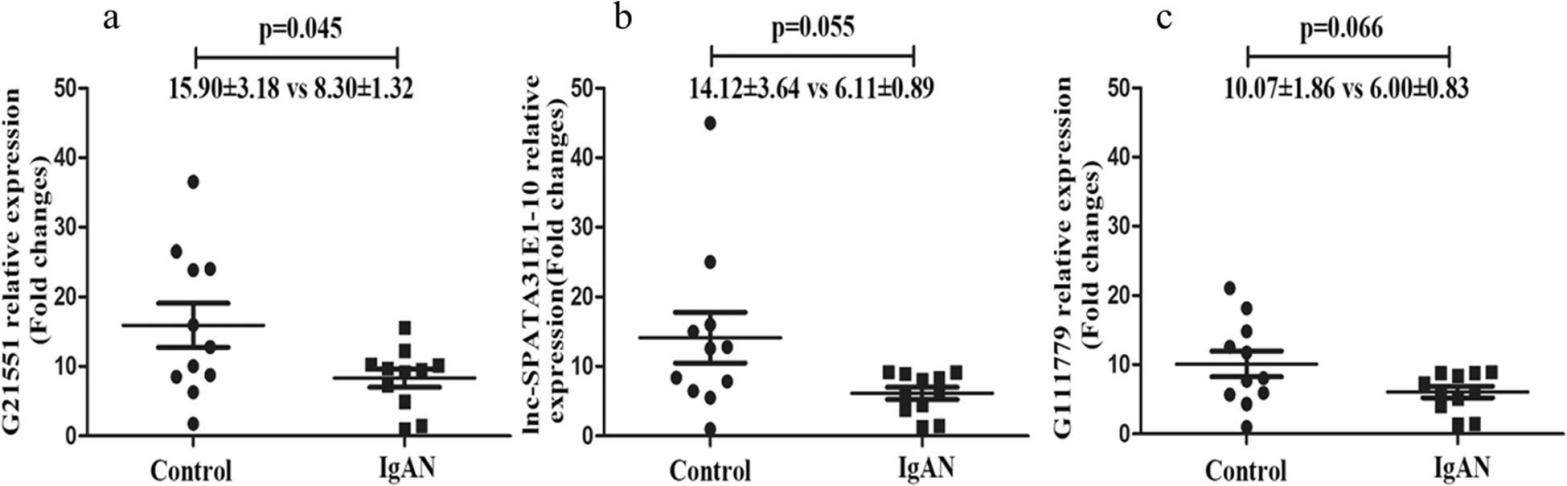
Validation of differentially expressed lncRNAs by qRT-PCR between IgAN patients and their healthy first-degree relatives. **a** lncRNA-G21551. **b** lnc-SPATA31E1–10. **c** lncRNA-G111779. Gene expression was calculated by the 2-ΔΔct method and normalized to external reference λpolyA+ RNA compared with the maximum ΔCt

### Validation of candidate lncRNAs by qRT-PCR and nearest protein-coding genes

To confirm the results obtained from high-throughput sequencing, ten candidate lncRNAs were selected from both up-regulated and down-regulated groups according to their base mean and |log_2_(FC)| (Table 2). qRT-PCR was performed on validation cohort (11 IgAN patients with their healthy relatives). Consistent with the sequencing results, lncRNA-G21551 was significantly down-regulated in patients with IgAN compared with their healthy relatives (8.30 ± 1.32 vs 15.90 ± 3.18 for IgAN patients vs control, *p* = 0.045) (Fig. 3a). Two other candidates, lnc-SPATA31E1–10 and lncRNA-G111779, were also down-regulated in patients with IgAN, but the difference was not statistically significant (6.11 ± 0.89 vs 14.12 ± 3.64, *p* = 0.055, 6.00 ± 0.83 vs 10.07 ± 1.86, *p* = 0.066 for IgAN patients vs control respectively) (Fig. 3b, c). The remaining 17 lncRNAs could not be validated by qRT-PCR due to their low abundance. FCGR3B was calculated to be the nearest protein-coding gene of lncRNA-G21551 using pathway enrichment analysis and visualization using the R package clusterProfiler. FCGR3B encodes for the low affinity receptor (FcgR3B receptor) of the Fc segment of immunoglobulin G (IgG) [21].

## Discussion

In this study, the exosomal lncRNA profiles of IgAN patients were measured and compared to their healthy first-degree relative. Through high-throughput RNA seq, we identified lncRNA-G21551 as a potential diagnostic biomarker for IgAN. We also predicted its potential role in IgAN pathogenesis through pathway enrichment analysis and visualization using R package clusterProfiler. The nearest protein-coding gene of lncRNA-G21551 was identified to be FCGR3B, which encodes for the low affinity receptor (FcgR3B receptor) of the Fc segment of immunoglobulin G (IgG). Until the pathogenesis of IgAN is elucidated, renal biopsy will remain the golden standard for the diagnosis of IgAN. Therefore, our finding may bring forward a clue to find the disease biomarkers by exosomal lncRNA profiles in IgAN. The establishment of differentially expressed exosomal lncRNA profiles in IgAN could be important for illustrating the pathogenesis of IgAN.

lncRNAs-based biomarkers have been reported in a variety of diseases including membranous nephropathy, IgAN and hepatocellular carcinoma [11, 13, 14]. Zuo et al. identified 167 differentially expressed lncRNAs (including 55 upregulated lncRNAs and 112 downregulated lncRNAs) in peripheral blood mononuclear cells (PBMCs) of IgAN, which may aid in the elucidation of a basic pathogenic mechanism [13]. However, unprotected lncRNAs are easily degraded by RNAse, and exosomes act as a protective layer that can prevent extracellular degradation of lncRNAs [14]. Recently, emerging studies on exosomal non-coding RNAs (including lncRNAs and miRNAs) in renal disease have been reported [22], but few reports has been focused on IgAN. Min et al. reported a significantly difference in urinary exosomal miRNA profiles (including miR-29c, miR-146a and miR-205) between IgAN and healthy controls, which may serve as novel biomarkers for IgAN [19]. However, the differential expression of lncRNAs in plasma exosomes in IgAN patients was not reported, which may contribute to the discovery of potential new biomarkers or pathogenic factors of IgAN from plasma, as the increased levels of circulatory polymeric IgA1 with aberrant O-glycosylation of its hinge region was reported to be the first-hit as of this disease [1].

In order to rule out the influence of genetic background, healthy first-degree relatives of the patients were used as normal controls. Through RNA-seq and qRT-PCR analysis, a large number of exosomal lncRNAs in the plasma were found differentially expressed between IgAN patients and their healthy first-degree relative, among them, the expression of lncRNA-G21551 were found to be significantly down-regulated in IgAN patients (8.298 ± 1.319 vs 15.896 ± 3.176 for IgAN patients vs control, *p* < 0.05), therefore, lncRNA-G21551 may serve as a biomarker for IgAN. However, the remaining 17 lncRNAs could not be validated by qRT-PCR due to their low abundance in exosomes. In our study, we used pathway enrichment analysis and visualization using the R package clusterProfiler and found that the nearest protein-coding gene of lncRNA-G21551 was FCGR3B, and hypothesize that lncRNA-G21551 may play a vital role in the pathogenesis of IgAN by regulating the expression of FCGR3B.

It was previously reported that copy number (CN) variation of the FCGR3B gene is associated with susceptibility to systemic lupus nephritis (SLE) and ANCA-associated systemic vasculitis (AASV) [23, 24]. Furthermore, the FcgR3B receptor is primarily expressed on neutrophils; while FCGR3B CNs are correlated with the expression of FcgR3B, functioning for the clearance of immune complex [25]. IgAN is frequently characterized by depositions of IgA (mainly IgA1) or IgA-containing immune complexes in the glomerular mesangial areas or the capillary wall. IgA1 deposits are usually detected along with complement component 3 (C3), and often with IgG or IgM or both in glomeruli [1]. However, a recent study has shown that FCGR3B polymorphisms have significant influence on the incidence and pathological grade of IgAN, suggesting that the impairment of IgG-IC clearance by the FCGR3B gene and subsequent glomerular deposition may also contribute to the glomerular lesions [26].

However, in this current study, we did not carry out mechanistic study on the direct evidence that could elucidate the interaction between lncRNA-G21551 and FCGR3B and the mechanisms involved in IgA nephropathy. The functional research of lncRNA-G21551 in IgAN may be an interesting new research area and is currently the scope of our research group.

## Conclusions

In summary, our study demonstrates a significant difference in plasma exosomal lncRNA expression profiles between IgAN patients and their first-degree relatives, providing novel information on the potential role of exosomal lncRNAs in IgAN. Pathway enrichment analysis and visualization reveals that the FCGR3B gene may be closely associated with the pathogenesis of IgAN. Therefore, the expression level of exosomal lncRNA-G21551 could be utilized as a promising biomarker for IgAN diagnosis.

## Methods

### Participants and sample selection

Patients with primary IgAN from Sun Yat-sen Memorial Hospital, Sun Yat-sen University (Guangzhou, China) between March 2018 and March 2019, and their first-degree relatives were recruited in this study. Inclusion criteria are as follows: 1) biopsy-proven IgAN (within 30 days prior to enrollment); 2) age ≥ 14 years; and 3) an adequate biopsy sample with ≥10 glomeruli. Exclusion criteria are as follows: 1) secondary IgAN (Henoch-Schönlein purpura, systemic lupus erythematosus, liver disease, etc.), 2) an estimated glomerular filtration rate (eGFR) < 30 mL/min/1.73 m^2^ (calculated by the Chronic Kidney Disease Epidemiology Collaboration [CKD-EPI] creatinine equation [27]; 3) prior treatment with RAAS (renin-angiotensin-aldosterone system) inhibitor and / or immunosuppressants drugs; 4) presence of diabetes, concomitant infections, severe metabolic syndrome, and malignant tumors. As this is a patient-control matched study, patients’ healthy first-degree relatives (parents, siblings, or children) were chosen as respective controls. For this study, a total of 17 patients and their first-degree relatives were recruited. This study was conducted according to the principles of the Declaration of Helsinki and was approved by the Ethical Review Committee of Sun Yat-sen Memorial Hospital, Sun Yat-sen University (SYSEC-KY-KS-2018-080). Written informed consent was obtained from all participants prior to the study.

Whole blood samples were collected from each participant using anticoagulant EDTA tubes and centrifuged at 3000×g, 4 °C for 10 min. Then, the supernatant was centrifuged at 15000×g under the same conditions. The plasma supernatant was stored immediately at − 80 °C until further analysis. Samples for RNA-Seq were obtained from 6 patients and 6 relatives. For validation, samples from other 11 patients with IgAN and 11 relatives were subjected to qRT-PCR to detect the expression level of ten candidate lncRNAs selected from both up- and down-regulated groups according to their base mean and |log_2_(FC)|. Demographic and baseline clinical data including gender, age, 24-h urinary protein excretion (UPE) and serum creatinine (sCr), blood urea nitrogen (BUN), Serum uric acid (UA) and eGFR were recorded at the time of kidney biopsy.

### Exosome isolation and identification

Exosomes form plasma of IgAN patients and their first-degree relatives were isolated using Exosome Precipitation Solution (ExoQuick-TC, System Biosciences, USA) according to the manufacturer’s instructions. Morphology of the isolated exosomes was then identified with Transmission Electron Microscope (TEM), size distribution analysis. Flow cytometer analysis was then use to confirm the purity of isolated exosomes.

### RNA extraction

After exosome extraction from serum, total RNAs were extracted from the17 patients and the corresponding relatives using miRNeasy Mini kit (Qiagen, Germany) individually according to the manufacturer’s instructions. Quantification of the total RNAs was performed by the Agilent 2200 TapeStation (Agilent Technologies, CA, USA).

### cDNA library construction and high-throughput RNA sequencing

For RNA-seq analysis, total RNA from the exosomes of the 6 IgAN patients and their corresponding relatives was used for library preparation and sequencing, which were performed at RiboBio (Guangzhou, China). Briefly, RNA was fragmented to approximately 200 bp. The individual RNA sample were then subjected to first and second strand cDNA synthesis followed by adaptor ligation and low-cycle PCR enrichment according to the instructions provided with the NEBNext® Ultra™ RNA Library Prep Kit for Illumina (NEB, USA). The purified library products were evaluated using the Agilent 2200 TapeStation and Qubit®2.0 (Life Technologies, USA) and then sequenced (2× 150 bp) using a HiSeq30000.

### Sequencing data analysis

To obtain high-quality, clean sequencing data, Fastp (version 0.19.4) [28] was used to filter low-quality reads, to cut adapters and for quality control of raw FASTQ files to obtain clean reads. The clean reads of each experiment were aligned against the human genome (UCSC/hg19) with HISAT2 (version 2.1.0) [29] and then subsequently assembled by StringTie (version 1.3.4d) [30] separately. All assemblies were merged into one transcriptome by TACO [31].

The newly assembled transcriptome was aligned to GENCODE v27 and Lncipedia v5.2 using GFFCompare (http://github.com/gpertea/gffcompare, version 0.10.1) to find novel transcripts which were assigned “class code” values of ‘i’, ‘u’ or ‘x’. The distances between novel transcripts and reference protein-coding transcripts were calculated by BED Tools (version 1.2.4) [32]. CPAT [33] and PLEK (version 1.2) [34] were used to calculate the coding potential of novel transcripts. Salmon (version 1.11.2) [35] was applied to quantify transcript expression.

To analyze differential gene expression of lncRNA, several correlative packages in R were used. Tximport (version 1.12.3) [36] was applied to import quantification of transcript expression in R. Then, differentially expressed genes were determined by DESeq2 (version 1.24.0) [37] using FDR 0.05 as the threshold, and ggplot2 [38] was used for visualization. Nearest protein-coding genes for differential lncRNAs were used to perform pathway enrichment analysis and visualization using the R package clusterProfiler [39].

### Quantitative real-time PCR (qRT-PCR) analysis

qRT-PCR was used to verify the RNA-Seq data. LncRNAs were chosen based on expression level and biological significance. Sixteen μL of total RNA was used to synthesize the first strand of cDNA using PrimeScript™ RT Master Mix (Catalog No. RR036A, Takara, Japan). Real-time PCR was performed using TB Green (Catalog No. RR420A, Takara, Japan) in 96-well plates using the Biorad CFX384 Real-Time System (Bio-Rad, CA). The relative levels of target exosome-packaged lncRNAs were normalized against a synthesized exogenous reference λ polyA+ RNA (Catalog No. 3789, Takara, Japan).

### Statistical analysis

Statistical analysis was performed with IBM SPSS Statistics 22.0 software (SPSS Inc., Chicago, IL, USA). Continuous data are expressed as mean ± standard deviation (S.D.). Data conforming to normal distribution were compared using Student t-test, while those with non-normally distributed were tested using Mann-Whitney U-test. Percentages (%) or frequencies was used for categorical data, and chi-squared test was used for comparison analysis between groups. *p* < 0.05 was considered statistically significant.

## Supplementary information

**Additional file 1.** Data source and Predicted sequence of lncRNA-G21551.

## Data Availability

Neither this manuscript nor substantial parts of it are under consideration for publication elsewhere, have been published nor made available elsewhere in a manner that could be construed as a prior or duplicate publication of the same content. The predicted sequence of lncRNA-G21551 was shown in supplementary data.

## Abbreviations

IgAN: Immunoglobulin A nephropathy
lncRNA: Long non-coding-RNA
FCGR3B: Fc-Gamma Receptor 3B
ESRD: End-stage renal disease
Gd-IgA1: Galactose-deficient IgA1
TfR: Transferrin receptor
DN: Diabetic nephropathy
MN: Membranous nephropathy
LN: Lupus nephritis
RA: Rheumatoid arthritis
SLE: Systemic lupus erythematosus
ELAVL1: ELAV like RNA binding protein 1
Xist: X-inactive Specific Transcrip
DEG: Differentially expressed genes
RNA-seq: RNA sequencing
qRT-PCR: Real-time quantitative polymerase chain reaction
IFN: Type-I interferon
eGFR: Estimated glomerular filtration rate
CKD-EPI: Chronic Kidney Disease Epidemiology Collaboration
UPE: Urinary protein excretion
sCr: Serum creatinine
BUN: Blood urea nitrogen
UA: Uric acid
TEM: Transmission Electron Microscope
IgG: Immunoglobulin G
PBMC: Peripheral blood mononuclear cell
CN: Copy number
AASV: ANCA-associated systemic vasculitis
BED Tools: Browser Extensible Data Tools
CPAT: Coding-Potential Assessment Tool
PLEK: Predictor of long non-coding RNAs and messenger RNAs based on an improved k-mer scheme
TACO: Transcriptome Assemblies Combined into One
