# Supplementary material for: Identification of differentially expressed circulating exosomal lncRNAs in IgA nephropathy patients
Source: BMC Immunol. 2020 Mar 31;21:16. doi: 10.1186/s12865-020-00344-1 (PMC7110735; doi:10.1186/s12865-020-00344-1)
Supplement: Supplementary file 1 — Additional file 1. Data source and Predicted sequence of lncRNA-G21551. [file 12865_2020_344_MOESM1_ESM.rtf]

Data source and Predicted sequence of lncRNA-G21551


Data source of lncRNA-G21551
The transcripts sequences in FASTA format were generated by Gffread utility (http://ccb.jhu.edu/software/stringtie/gff.shtml#gffread) with human genome (UCSC/hg19) and our newly assembled trnanscritome.

Predicted sequence of lncRNA-G21551

>TU27485 gene=G21551 loc:chr1|161586283-161587128|- exons:161586283-161586506,161586663-161587128 segs:1-466,467-690
tgcccagccgaagctgcactgtgctgctgccatctcggcttactgcaacctacctgcctgattctcctgcctcagcctgccgagtgcctgcgattgcaggcgcacgccgccacgcctgactggttttcgtatttttttggtggagacggggtttcgccgtgttggccgtgctggtctccagctcctaaccgcgagtgatctgccagcctcggcctcccaaggtgccgggattgcagacggagtctcgttcactcagtgctcaatgttgcccaggctggagtgcagtggcgtggtctcggctagctacaacctccacctcccagccgcctgacttggcctcccaaagtgccgagattgcagcctctgcccggctgccaccccgtctgggaagtgaggagcgtctctgcctggcctcccatcgtctgggatgtgaggagcccctctgcccggctgcccagtctgggaagtgaggagcgtctctgcccagccgccccgtctgagaagtgaggagcccctccgcccggcagccgccccgtctgggaagtgaggagcgcctctgcccggcagccaccccatctgggagggagatggggggcgactctgccctgccaccgccccgtccgggaggtggggggtgcctctgcccgtctgccccttctgggaagtgaggagcccctctgcccagccgcc
